# Supplementary material for: Ultrasensitive Detection of Cu(II) and Pb(II) Using a Water-Soluble Perylene Probe
Source: Molecules. 2022 Oct 20;27(20):7079. doi: 10.3390/molecules27207079 (PMC9608940; doi:10.3390/molecules27207079)
Supplement: Supplementary file 1 [file molecules-27-07079-s001.zip › molecules-1943236-supplementary.pdf]

SUPPORTING INFORMATION

Ultrasensitive detection of Cu(II) and Pb(II) using a water soluble perylene probe

Erika Kozma[1*], Antonella Caterina Boccia[1], Anita Andicsova-Eckstein[2], Alfio Pulvirenti[1], Chiara Botta[1*]

[1] Consiglio Nazionale delle Ricerche, Istituto per le Scienze e Tecnologie Chimiche 'Giulio Natta', Via Alfonso Corti 12, 20133 Milano, Italy

[2]Slovak Academy of Sciences, Polymer Institute, Dubravska Cesta 9, Bratislava, Slovakia

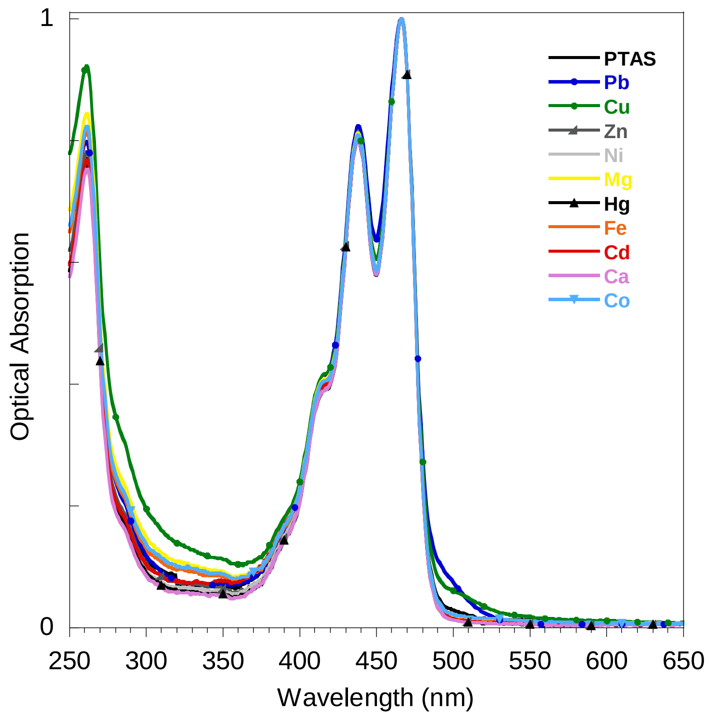

Figure S1a. Normalized absorption spectra of PTAS 10μM water solutions upon addition of 10 μM metal ions

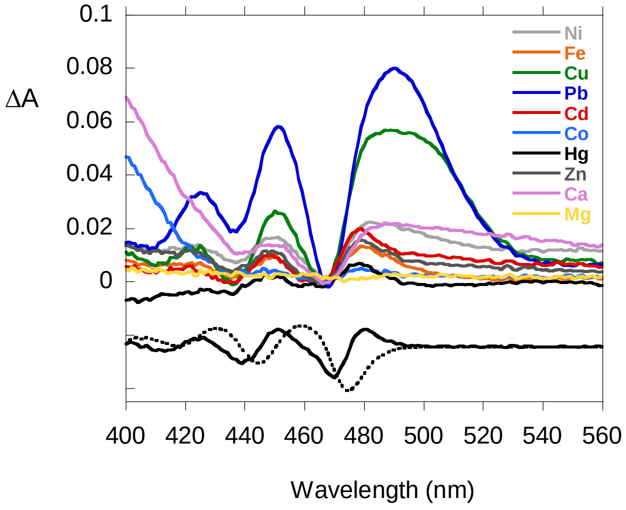

Figure S1b. Absorbance variations (spectra Fig.S1a) compared to the first (dotted line) and second (solid line) derivative of PTAS absorption spectrum.

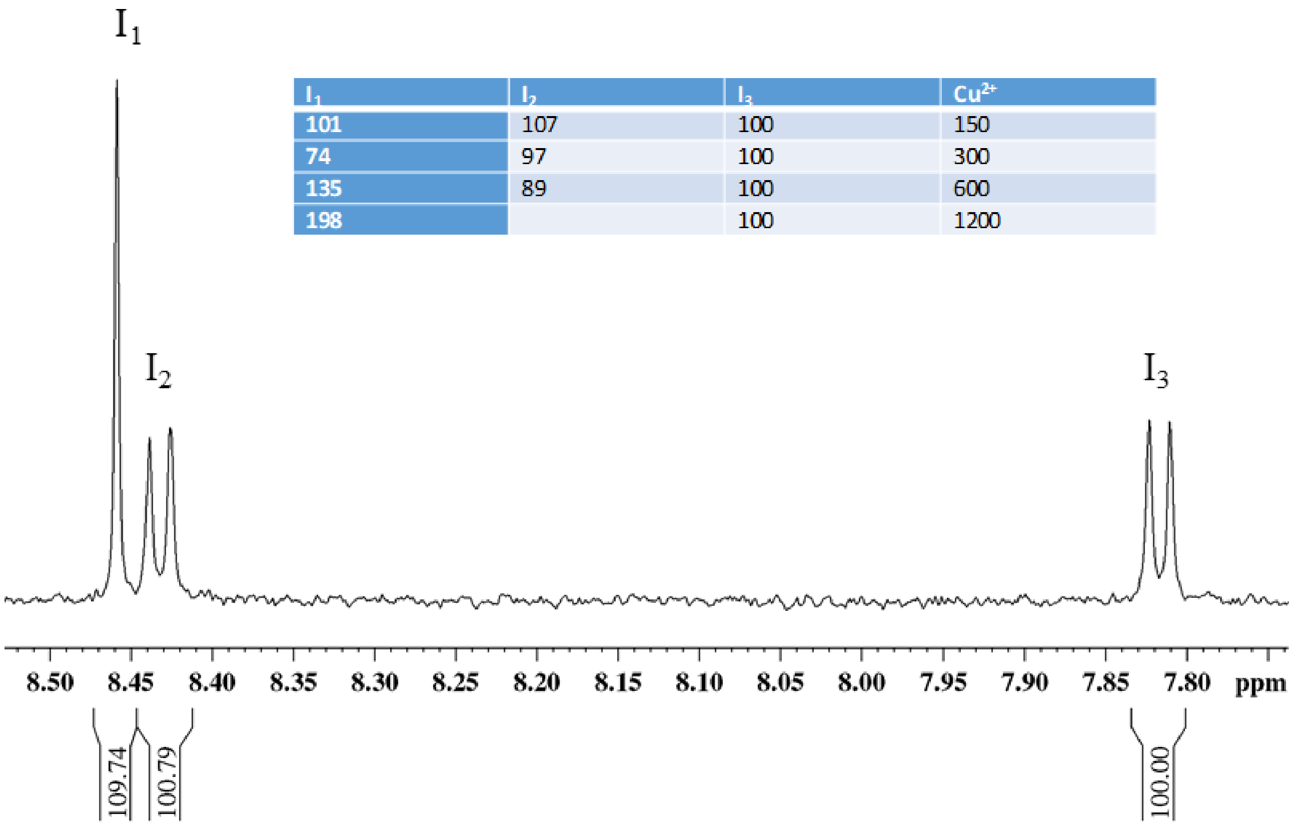

I_1	I_2	I_3	Cu^{2+}
101	107	100	150
74	97	100	300
135	89	100	600
198		100	1200

Figure S2. ^{1}H NMR spectrum, with integrals value, of 30 µM PTAS and 15 µM Cu^{2+} solution (PTAS:Cu^{2+}=2:1), in D_2O, at 600 MHz. In the table are reported the integrals value referring to the titration experiments and evaluated at several PTAS:Cu^{2+} ratios.

Table S1. Sensor performances of perylene-based probes for the detection of lead and copper ions

PDI	Sensing mechanism	Detection method	PDI conc.	Solution	Analyte	Detection Limit	Ref.
DPPCA-Au NPs	displacement of AuNPs with Cu^{2+}	Fluorescence off-on	1 µM	$CHCl_3$/acetonitrile	Cu^{2+}	10^{-6}M	Adv. Mater. 2005, 17, 2811
PDI-DPEN	Metal coordination	Colorimetric, Fluorescence on-off	10 µM	H_2O/THF	Cu^{2+}	10^{-5}M	Sci. in China Series B: Chem. 2009, 52, 518
PDI-3	Metal coordination	Colorimetric, Fluorescence on-off	10 µM	$CHCl_3$	Cu^{2+}	10^{-6} M	J. of Fluorescence 2014, 24, 909
PDI-L-DOPA	Metal coordination	Colorimetric, Fluorescence off-on	0.05 µM	Acetonitrile	Cu^{2+}	10 µM	ACS Appl. Mater. Interfaces 2014, 6, 23, 21369
PAM-PBI	Metal coordination	Colorimetric, Fluorescence on-off	10 µM	THF/MOPS buffer	Cu^{2+}	0.17 mM	Tet. Lett. 2014, 55 (21), 3218
PTCDI	Metal coordination	Fluorescence off-on	5 mM	Water/aptamer	Pb^{2+}	0.1 ng·mL^{-1}	Microchim. Acta 2017, 184, 2439
PTAS	Metal coordination	Optical response (absorption mode)	30 µM	Water	Cu^{2+}, Pb^{2+}	2 µM	This work
